# Clinical Determinants of Halitosis in Elderly Patients with Complete, Partial, and Fixed Prosthetic Rehabilitation

**DOI:** 10.3390/jcm15124590

**Published:** 2026-06-12

**Authors:** Romina Georgiana Bita, Otilia Cornelia Boloș, Edida Maghet, Adrian Boloș, Raluca Briceag, Bogdan Andrei Bumbu

**Affiliations:** 1 2nd Department of Radiology and Medical Imaging, General and Dento-Maxillary Imaging, Dental Medicine Faculty, “Victor Babeș” University of Medicine and Pharmacy, 300041 Timișoara, Romania; romina.bita@umft.ro; 2 Department of Dental Aesthetics, Faculty of Dental Medicine, “Victor Babeș” University of Medicine and Pharmacy, 300041 Timișoara, Romania; bolos.otilia@umft.ro; 3 Faculty of Dental Medicine, “Victor Babeș” University of Medicine and Pharmacy, 300041 Timișoara, Romania; edida.maghet@umft.ro; 4 Department of Oral Rehabilitation, Faculty of Dental Medicine, Specialization of Dental Technology, “Victor Babeș” University of Medicine and Pharmacy, 300041 Timișoara, Romania; 5 Faculty of Dental Medicine, Ovidius University of Constanța, 900684 Constanța, Romania; 6 Department of Dental Medicine, Faculty of Medicine and Pharmacy, University of Oradea, 410073 Oradea, Romania; bogdanbumbu@uoradea.ro

**Keywords:** halitosis, aged, dental prosthesis, polypharmacy, xerostomia

## Abstract

**Background/Objectives**: Halitosis in geriatric patients is multifactorial, but the joint contribution of prosthetic rehabilitation type and polypharmacy after routine dental procedures has rarely been quantified. We investigated how prosthesis type, polypharmacy, and salivary function were associated with volatile sulfur compound (VSC) burden and self-perceived halitosis in elderly dental patients. **Methods**: This cross-sectional study enrolled 88 patients aged ≥65 years, four weeks after completing routine dental procedures. Participants were stratified into three groups: complete denture wearers (*n* = 30), partial removable denture wearers (*n* = 28), and fixed prostheses/implants (*n* = 30). We measured unstimulated salivary flow rate (uSFR), tongue coating index (TCI), denture biofilm index, total VSCs (Halimeter^®^), organoleptic score (0–5), and self-perceived halitosis. Polypharmacy, comorbidities, and the Geriatric Oral Health Assessment Index (GOHAI) were recorded. Analyses included one- and two-way ANOVA, Spearman correlations, theory-informed multivariable linear and logistic regression, exploratory mediation analysis, and ROC curves. **Results**: Forty-two participants (47.7%) reported halitosis. Mean VSC differed across groups (complete dentures 278.2 ± 38.6 ppb; partial 211.2 ± 46.3 ppb; fixed 164.4 ± 43.9 ppb; ANOVA *p* < 0.001). uSFR correlated inversely with VSC (ρ = −0.61, *p* < 0.001) and TCI correlated positively (ρ = 0.56, *p* < 0.001). A significant prosthesis × polypharmacy interaction was observed (F = 3.74, *p* = 0.029, η^2^*_p_* = 0.082): polypharmacy was associated with higher VSC most clearly among partial and fixed prostheses wearers, whereas complete denture wearers showed high VSC levels regardless of polypharmacy status. Exploratory mediation findings were consistent with partial indirect association, with 45.9% of the polypharmacy–VSC association statistically explained by reduced uSFR; however, the cross-sectional design precludes causal or temporal interpretation. The full multivariable model showed apparent discrimination for self-perceived halitosis (AUC = 0.92), while the simplified four-item chairside composite model showed AUC = 0.89; neither estimate was optimism-corrected or externally validated. **Conclusions**: In elderly post-procedure patients, complete denture wearing, polypharmacy, and salivary hypofunction were independently and jointly associated with higher halitosis burden. Reduced salivary flow was consistent with a partial indirect statistical pathway in the polypharmacy–VSC association, supporting hydration counseling and meticulous prosthesis hygiene as low-cost geriatric interventions. Sensitivity analyses excluding implant-supported restorations, participants with MMSE scores of 24–26, and expanded mediation models including TCI and biofilm/plaque did not materially change the main inference.

## 1. Introduction

Halitosis is one of the most common oral health complaints among older adults, with self-reported prevalence ranging between 24% and 51% in patients above 65 years of age. Although the literature has long established its multifactorial origin, geriatric halitosis differs from the adult presentation in several clinically important ways: it more often coexists with reduced salivary flow, polypharmacy-induced xerostomia, denture-related biofilms, and a generally compromised oral environment. These layered determinants mean that elderly patients can experience persistent malodor even after they have apparently completed routine dental treatment, and the resulting social withdrawal can compound the loneliness already common in this age group [1,2,3].

Routine dental procedures performed in elderly patients (such as periodontal therapy, denture relining, partial denture adjustment, crown cementation, or implant restoration) modify the local ecology in different ways. Removable denture interfaces remain a particularly hospitable niche for sulfide-producing anaerobes such as *Porphyromonas* spp., *Prevotella* spp., and *Solobacterium moorei*; their biofilms can re-establish within days of cleaning if hygiene compliance is suboptimal. Fixed prostheses, in contrast, are easier to clean, but micro-gaps at restoration margins and around implants can still harbor odorigenic species [4,5]. The relative magnitude of these post-procedure halitosis risks across prosthesis types is not well characterized in older adults.

Salivary hypofunction is the second pillar of geriatric halitosis. Healthy unstimulated whole-saliva flow rate (uSFR) is conventionally ≥0.30 mL·min^−1^, but in patients above 70 years of age, mean values frequently fall below 0.20 mL·min^−1^, both because of glandular involution and because of the cumulative xerogenic burden of chronic medications. Anticholinergics, antihypertensives, antidepressants, antihistamines, diuretics, and proton pump inhibitors are all over-represented in geriatric prescriptions and are independently associated with reduced flow. Saliva normally dilutes precursors of volatile sulfur compounds (VSCs), buffers acidic pH, and delivers antimicrobial peptides; its loss therefore disinhibits VSC generation [6,7,8,9].

Polypharmacy, generally defined as the concomitant use of five or more medications, is reported in 30–60% of community-dwelling older adults and in over 80% of nursing home residents. Beyond xerostomia, polypharmacy reflects accumulated comorbidity (diabetes, hypertension, depression, COPD), each of which can independently alter oral ecology. Despite these clear linkages, very few studies have tried to separate the association of polypharmacy with halitosis from the portion of that association statistically explained by reduced salivary secretion. Exploratory mediation analysis is a useful descriptive framework for this question, but in a cross-sectional design it should be interpreted as an indirect statistical association rather than evidence that the exposure precedes the mediator or outcome [10,11,12].

Self-perceived halitosis adds an additional layer of complexity. Older adults are sometimes unaware of their own malodor because of olfactory adaptation and age-related anosmia, while in other cases halitophobia leads to overestimation of the problem. Validated objective measures (Halimeter^®^ for total VSCs and organoleptic scoring by trained panelists) therefore must be combined with structured self-report to obtain a clinically meaningful picture. The Geriatric Oral Health Assessment Index (GOHAI) further captures the impact of oral malodor on quality of life, eating, and social interaction, and its inclusion alongside biochemical metrics gives a more rounded view of the patient’s experience [13,14,15,16,17,18,19,20,21].

The present cross-sectional study addresses this gap by examining halitosis four weeks after routine dental procedures in 88 community-dwelling adults aged ≥65 years, stratified by the type of prosthetic rehabilitation they received. We aimed to (i) compare halitosis indicators across prosthesis types; (ii) assess whether the association between polypharmacy and VSC burden differed according to prosthesis type; (iii) explore the proportion of the polypharmacy–VSC association statistically explained by reduced uSFR; and (iv) build exploratory, internally unvalidated clinical screening models for self-perceived halitosis [22,23,24,25,26,27,28,29,30]. By integrating biochemical, behavioral, and quality-of-life dimensions in the same elderly cohort, we hope to deliver evidence-based, low-cost recommendations for the dental team caring for older patients, while recognizing that the observational design cannot establish causality.

## 2. Materials and Methods

### 2.1. Study Design and Ethical Considerations

This was an observational, analytical cross-sectional study conducted between September 2023 and June 2024 at the geriatric dental outpatient unit of the Faculty of Dental Medicine, “Victor Babeș” University of Medicine and Pharmacy Timișoara. The protocol was developed in accordance with the Declaration of Helsinki and reviewed and approved by the Institutional Ethics Committee (approval code GD-2023-114, dated 12 July 2023). Reporting follows the Strengthening the Reporting of Observational Studies in Epidemiology (STROBE) checklist for cross-sectional studies, and the analytical plan was pre-registered before data collection began. The PICO framework was defined as follows: the population comprised community-dwelling adults aged 65 years or older who had completed a routine dental procedure within the four weeks preceding assessment; the exposures of interest were prosthetic rehabilitation type, polypharmacy status (≥5 chronic medications), and unstimulated salivary flow rate; the comparators were patients with different rehabilitation types, with or without polypharmacy, and with higher versus lower uSFR; and the outcomes were objective halitosis indicators (total VSC, organoleptic score) and self-perceived halitosis.

All participants provided written informed consent prior to enrolment; for individuals with mild cognitive impairment (Mini-Mental State Examination [MMSE] score 24–26), consent was countersigned by an accompanying caregiver. The procedures were entirely non-invasive (passive saliva collection, intraoral photograph, breath sampling, structured questionnaire) and no adverse events were reported. Personal identifiers were replaced by sequential numerical study IDs at the point of data entry, and the de-identified dataset was held on an encrypted institutional server with access limited to the principal investigator and the data manager. The study was conducted in compliance with the General Data Protection Regulation (GDPR), and participants retained the right to withdraw their data at any point. No financial compensation was offered, but participants received a complimentary oral hygiene kit and a written report of their breath assessment. The article processing charge will be covered by institutional research funds; no external sponsor influenced study design, conduct, or reporting. Ten participants (11.4%) had MMSE scores between 24 and 26; all were accompanied by a caregiver, and a sensitivity analysis excluding them was added to assess the robustness of self-reported outcomes.

### 2.2. Participants and Recruitment

Eligible patients were consecutively invited from the geriatric dental outpatient list during their routine four-week post-procedure follow-up visit. Inclusion criteria were: (i) age ≥ 65 years; (ii) completion of one of the three index procedures within the previous 28 ± 4 days (complete denture relining and occlusal adjustment; partial removable denture adjustment with abutment tooth cleaning; or fixed prosthesis/implant crown cementation); (iii) ability to attend an early-morning study visit; and (iv) MMSE score ≥ 24 to ensure reliable self-report. Exclusion criteria comprised: active upper-respiratory infection in the previous two weeks; antibiotic, antifungal, or antiseptic mouthwash use in the previous four weeks; head-and-neck radiotherapy or chemotherapy in the previous 12 months; uncontrolled Sjögren’s syndrome or other primary salivary gland disease; active untreated dental caries or probing pocket depths > 5 mm at the assessment visit; current smoking >10 cigarettes per day; nasogastric or gastrostomy feeding; and inability to provide informed consent. Patients undergoing surgical implant placement or any active osseointegration period were not included; implant-related cases in the fixed/implant group referred only to definitive crown cementation or restoration on implants already confirmed as osseointegrated for at least three months.

An a priori sample size calculation (G*Power 3.1.9.7) for the primary aim of detecting a between-group difference in mean log_10_-VSC across three prosthesis groups, assuming f = 0.40 (large effect size), α = 0.05, and 1 − β = 0.85, indicated 84 participants. Allowing for 10% attrition or unusable data, a target enrolment of 92 was set. Of 104 patients screened, 12 were excluded (5 because of recent antibiotic use, 4 because of active respiratory infection, and 3 declined participation), and 92 were enrolled. Four participants were lost between recruitment and data collection (rescheduling failures and one acute illness) so that the final analytical dataset comprised 88 participants. Allocation to the three analytical groups was determined by the index procedure: complete denture wearers (Group A, *n* = 30), partial removable denture wearers (Group B, *n* = 28), and fixed prosthesis/implant wearers (Group C, *n* = 30). Both authors involved in clinical assessments (a calibrated periodontist and a calibrated prosthodontist) were blinded to self-reported halitosis status during breath examination. The 28 ± 4-day visit was selected to capture routine post-treatment status after the immediate procedural phase, while recognizing that plaque and biofilm can re-establish within days; therefore, sensitivity analyses were added to test whether the main findings persisted after excluding implant-supported restorations.

### 2.3. Clinical Examination, Sampling Standards, and Variables

All assessments were performed in a single early-morning session (07:30–10:00 a.m.) in a temperature-controlled (22 ± 1 °C) operatory. Participants were instructed to refrain from food, drinks other than still water, oral hygiene practices, denture cleaning, chewing gum, smoking, and the use of perfumed personal care products from midnight until the examination. Compliance was verbally checked on arrival; non-compliant participants were rescheduled. The following sequence was followed to minimize cross-contamination of measurements: (i) structured questionnaire and self-perceived halitosis item; (ii) unstimulated saliva collection by passive drool over five minutes into pre-weighed low-evaporation polypropylene tubes (uSFR expressed in mL·min^−1^); (iii) two-minute mouth rest interval; (iv) total VSC measurement using portable sulfide monitor (Halimeter^®^, Interscan Corp., Los Angeles, CA, USA), expressed in parts-per-billion (ppb); (v) organoleptic scoring on the Rosenberg 0–5 scale by two calibrated examiners blinded to all other data, with consensus reached if scores differed by more than one point (weighted Cohen’s κ = 0.84); (vi) intraoral examination with tongue coating index (TCI) on the modified Winkel scale (six dorsal sextants scored 0–3, mean reported); and (vii) prosthesis or dentition examination, including denture biofilm index for removable prosthesis wearers (Augsburger–Elahi index, 0–4) or supragingival plaque index for fixed prosthesis wearers (Silness–Löe, 0–3).

Halimeter^®^ measurement protocol. Total VSC was measured according to the Interscan Halimeter RH17K manufacturer protocol [31,32,33]. Before sampling, participants kept the mouth closed for 3 min and refrained from speaking; during sampling they breathed quietly through the nose, did not blow or suck through the sampling straw, and no nasal clip was used. A new disposable straw was inserted approximately 2–3 cm into the slightly opened mouth without lip closure around the straw. The instrument was zero-calibrated to ambient air before each measurement session and checked according to the manufacturer schedule. Three readings were obtained at 60 s intervals, and the mean of the two closest readings was used for analysis; when the three readings differed by more than 20 ppb, a fourth reading was obtained and the two closest values were averaged.

Biofilm/plaque definitions. For removable prostheses, the denture biofilm score followed the Augsburger–Elahi denture plaque index after disclosing solution application, with visible plaque/debris graded from 0 (no visible plaque) to 4 (very heavy plaque or confluent deposits) on the tissue-contacting and polished surfaces; the mean score was used [31]. For fixed prostheses and implant crowns, plaque was recorded at crown/abutment margins using the Silness–Löe plaque index from 0 (no plaque) to 3 (abundant plaque visible to the naked eye) [32]. This combined variable was labeled “denture biofilm/plaque” because the relevant surface differed by rehabilitation type.

Covariates collected through a structured interview and chart review included: age, sex, body mass index, number of chronic medications taken daily for at least the previous three months, polypharmacy status (≥5 medications, dichotomous), specific xerogenic medication use (anticholinergics, tricyclic antidepressants, diuretics, antihistamines), self-reported physician-diagnosed diabetes mellitus, smoking history, mouth breathing during sleep (validated single-item Sleep Breathing Questionnaire), nocturnal denture wear (for removable prosthesis groups), denture cleaning frequency (daily versus less than daily), and number of remaining natural teeth. Quality of life was assessed using the 12-item Geriatric Oral Health Assessment Index (GOHAI; range 12–60, lower scores indicating worse oral health-related quality of life). Functional status was captured with the Katz Activities of Daily Living scale (range 0–6), and a binary variable indicating any functional dependence (Katz ≤ 1) was derived for subgroup analysis. The structured questionnaire was administered face-to-face by a single trained interviewer to minimize self-report variability. All instruments and intraoral measurements had been calibrated against a senior reference examiner during a pilot phase of 15 patients in the four weeks preceding study commencement. A xerogenic medication count was additionally calculated by summing daily medications with recognized salivary adverse effects, including anticholinergics, antidepressants, antihistamines, diuretics, beta blockers, calcium channel blockers, anxiolytics/sedatives, and proton pump inhibitors; this count was analyzed as a sensitivity alternative to the binary polypharmacy variable. Tongue cleaning behavior, denture adhesive use, and the specific type of denture cleaning solution were not recorded systematically and were therefore addressed as limitations rather than regression covariates.

### 2.4. Statistical Analysis

The analyses were performed in IBM SPSS Statistics version 29.0 (IBM Corp., Armonk, NY, USA), R version 4.3.1 (R Foundation for Statistical Computing, Vienna, Austria) for the mediation analysis (R package “lavaan” and “mediation”), and Python 3.11 for figure preparation. Distributional assumptions were assessed through Shapiro–Wilk testing for normality and through Levene’s test for homogeneity of variance. Skewed variables (total VSC, organoleptic score, number of medications) were log_10_-transformed for parametric analyses, with non-parametric alternatives (Mann–Whitney U, Kruskal–Wallis) used to confirm robustness. Continuous data are presented as mean ± standard deviation and categorical data as frequency (percentage). Between-group comparisons across the three prosthesis groups used one-way ANOVA with Tukey’s honest significant difference post hoc test for continuous variables, and Pearson chi-square or Fisher’s exact test for categorical variables. Effect sizes were reported as partial η^2^ or Cohen’s d. Spearman rank correlations were calculated for bivariate associations among the principal continuous variables, with Bonferroni-corrected significance thresholds applied to the matrix.

To address the main hypothesis of effect modification, a 3 (prosthesis type) × 2 (polypharmacy) two-way ANOVA on log_10_-VSC tested the main effects and the prosthesis × polypharmacy interaction. Predictors of log_10_-VSC were evaluated using a theory-informed multivariable linear regression model. Variables were selected a priori based on clinical plausibility and prior literature rather than automatic stepwise selection, and included uSFR, TCI, polypharmacy, mouth breathing, number of remaining teeth, denture biofilm/plaque, age, and sex; multicollinearity was excluded by variance inflation factors below 2.0. The determinants of self-perceived halitosis were examined through a multivariable logistic regression; model fit was evaluated using the Hosmer–Lemeshow test and the Nagelkerke pseudo-R^2^. Because the logistic model included eight predictors for 42 outcome events, the events-per-variable ratio was approximately 5.3, and odds ratios and discrimination metrics were interpreted as exploratory and potentially optimistic. No optimism correction, k-fold cross-validation, shrinkage procedure, or independent external validation sample was available. Discrimination of the full multivariable model and of a simplified four-item chairside composite was compared by receiver operating characteristic (ROC) analysis with bootstrap 95% confidence intervals (1000 resamples) and DeLong’s test. Mediation analysis was performed with 5000 bootstrap resamples to explore whether the association between polypharmacy and VSC was statistically explained by uSFR, following the conditional process framework of Hayes [15]. For both the primary and sensitivity mediation models, bootstrap confidence intervals were calculated for the indirect effect and for the proportion explained, with the proportion defined as the indirect effect divided by the total effect on the log10-VSC scale. Because exposure, mediator, and outcome were measured at the same visit, mediation terms are interpreted as indirect statistical associations rather than proof of temporal or causal pathways. All tests were two-tailed with α = 0.05; missing values were rare (<1.2%) and handled by listwise deletion after confirming the missing completely at random pattern with Little’s test (*p* = 0.59). Additional sensitivity analyses were then performed: (i) excluding implant-supported restorations from the fixed/implant group; (ii) excluding participants with MMSE scores of 24–26; (iii) repeating the mediation model after adding TCI and denture biofilm/plaque to the original covariate set; and (iv) replacing binary polypharmacy with the xerogenic medication count. TCI and biofilm/plaque were not included in the primary mediation model because they may represent parallel oral biofilm pathways or downstream correlates of low salivary clearance; they were therefore evaluated in an expanded sensitivity model to avoid overadjustment in the primary analysis. For the MMSE sensitivity analysis, the self-perceived halitosis logistic model was also re-estimated after excluding these participants and the direction and approximate magnitude of key adjusted odds ratios were compared with the primary model, rather than reporting only the interaction *p*-value.

## 3. Results

Complete denture wearers were on average 6.7 years older than fixed prosthesis wearers (76.4 vs. 69.7 years, ANOVA *p* < 0.001) and carried a heavier medication burden (6.3 vs. 3.4 daily medications, *p* < 0.001), with seven out of 10 meeting criteria for polypharmacy compared with only 36.7% in the fixed prosthesis group (chi-square *p* = 0.034). Mouth breathing during sleep showed a parallel descending gradient (40.0% vs. 25.0% vs. 13.3%; *p* = 0.043), as did functional dependence (30.0% vs. 14.3% vs. 6.7%; *p* = 0.039). Although sex distribution and BMI were comparable across groups (*p* > 0.6), oral health metrics diverged sharply: unstimulated salivary flow declined from 0.28 mL·min^−1^ in fixed prosthesis wearers to 0.18 mL·min^−1^ in complete denture wearers (*p* < 0.001), while tongue coating, denture biofilm/plaque, total VSCs, and organoleptic score moved in the opposite direction. Notably, mean VSC of 278.2 ppb in complete denture wearers exceeded the conventional clinical threshold for socially perceptible halitosis (~200 ppb) by 39%, whereas the fixed prosthesis mean of 164.4 ppb sat comfortably below it. These baseline imbalances are important for interpretation: prosthesis type in this cohort also indexed broader differences in age, medication burden, functional dependence, remaining teeth, and oral environment status, and therefore should not be interpreted as an isolated causal exposure. The xerogenic medication count paralleled total medication burden (2.2 ± 1.1 in complete denture wearers, 1.5 ± 0.9 in partial denture wearers, and 1.1 ± 0.8 in fixed/implant wearers; *p* = 0.001), supporting the decision to test xerogenic count as a medication-specific sensitivity variable. Ten patients had MMSE scores of 24–26 and were retained in the primary analysis with caregiver-countersigned consent (Table 1).

Table 2 maps the bivariate architecture of geriatric halitosis and uncovers several biologically coherent gradients. Unstimulated salivary flow emerged as the strongest single correlate of total VSC (ρ = −0.61, *p* < 0.001), implying that lower basal flow was associated with higher sulfur gas concentration. Tongue coating burden showed the second strongest VSC association (ρ = 0.56, *p* < 0.001) and was itself inversely linked to salivary flow (ρ = −0.49), suggesting that diminished clearance may coexist with the accumulation of desquamated cells and bacteria on the dorsum. Notably, the number of chronic medications correlated positively with VSC (ρ = 0.49, *p* < 0.001) and negatively with uSFR (ρ = −0.42), establishing the bivariate framework for the exploratory mediation analysis presented later. The denture biofilm/plaque index was significantly associated with VSC (ρ = 0.47), but was less strongly correlated with salivary flow (ρ = −0.36), indicating partial independence from the saliva–coating axis. Number of remaining teeth was protective in the expected direction (ρ = −0.39 with VSC), and GOHAI scores degraded as VSC rose (ρ = −0.51), confirming that objective sulfur burden was reflected in the patient’s oral-health-related quality of life. The organoleptic score correlated very strongly with instrumental VSCs (ρ = 0.71), supporting convergence between the panelist and instrumental olfactory assessments.

Table 3 shows an unadjusted monotonic gradient of halitosis severity across prosthesis types, with large between-group effect sizes. The Tukey post hoc test demonstrates that every pairwise comparison reached statistical significance for total VSC, log_10_-VSC, organoleptic score, and tongue coating index. Cohen’s d values of 2.74 (complete vs. fixed), 1.56 (complete vs. partial), and 1.04 (partial vs. fixed) for VSC all exceed the threshold of 0.8 conventionally classed as “large.” The progression from 278.2 ppb in complete denture wearers to 164.4 ppb in fixed prosthesis wearers represents a 41% reduction in absolute sulfur output, sufficient to move group means from above to below the social acceptability threshold (~200 ppb). The parallel decline in organoleptic ratings (3.4 to 1.9 points on the 0–5 scale) confirms that this difference is perceptible to trained examiners. However, because prosthesis groups differed in age, medication burden, functional dependence, and remaining dentition, these gradients should be interpreted as adjusted only in subsequent multivariable models and not as proof that prosthesis type alone caused the observed VSC differences.

Table 4 summarizes a theory-informed multivariable model in which clinical variables jointly explained 62% of the inter-individual variation in log_10_-VSC. Salivary flow showed the strongest independent association: each 0.1 mL·min^−1^ decrease in uSFR corresponded to a 0.46-log_10_ higher VSC value, equivalent to roughly a 35% higher ppb level at the cohort mean. Tongue coating retained a substantial independent association (β = 0.28, *p* < 0.001), consistent with the dorsum biofilm acting as an ecological niche partially separable from salivary clearance. Polypharmacy remained positively associated with VSC after adjustment (β = 0.21, *p* = 0.004), suggesting that the medication–halitosis association was not entirely captured by reduced saliva, since uSFR was already included in the model. Mouth breathing, denture biofilm/plaque, and number of remaining teeth all contributed modest yet statistically significant additional variance, in directions consistent with biological expectation. Age did not reach statistical significance once proximal variables were controlled (β = 0.08, *p* = 0.187), supporting the interpretation that chronological age may operate largely through medication burden, functional status, and oral environment changes. Sex was likewise non-significant. The model should be interpreted as explanatory and hypothesis-generating rather than as a stable predictive equation, because it was not validated in an independent cohort.

Mann–Whitney U or independent samples *t*-test was used as appropriate; chi-square was used for categorical variables. The denture cleaning subgroup includes only complete and partial denture wearers (*n* = 58).

Table 5 isolates two behavioral exposures that often go un-asked in routine geriatric dental visits and shows that both have outsize effects on halitosis severity. Among the 23 patients who reported sleep mouth breathing, mean unstimulated salivary flow was 32% lower than in nasal breathers (0.17 vs. 0.25 mL·min^−1^, *p* < 0.001), and total VSC was approximately 30% higher (262.7 vs. 201.4 ppb, *p* < 0.001). Tongue coating, denture biofilm, and organoleptic ratings all moved in the same direction, with effect sizes in the moderate-to-large range (Cohen’s d = 1.13 for VSC, 1.16 for organoleptic). The proportion of mouth breathers reporting subjective halitosis (69.6%) was nearly double that of nasal breathers (40.0%, chi-square *p* = 0.014). The right-hand panel of the table, restricted to the 58 denture wearers, shows an even larger and arguably more clinically actionable contrast: patients who cleaned their dentures less than once daily had a mean VSC of 274.6 ppb compared with 218.4 ppb in daily cleaners (*p* < 0.001), and their denture biofilm index was 69% higher. Although uSFR did not differ significantly between cleaning subgroups (*p* = 0.083), tongue coating did (1.8 vs. 2.3, *p* = 0.002), suggesting that poor denture hygiene seeds a broader oral cavity colonization rather than acting purely on the prosthesis surface.

Nagelkerke R^2^ = 0.58; Hosmer–Lemeshow goodness-of-fit χ^2^ = 6.12, df = 8, *p* = 0.634; correctly classified 81.8% of cases. Because there were 42 outcome events and eight predictors, the events-per-variable ratio was approximately 5.3; coefficients and classification performance should therefore be interpreted cautiously.

Table 6 quantifies how each predictor was associated with the patient’s subjective awareness of bad breath, and the magnitudes are clinically meaningful. A 0.1 mL·min^−1^ rise in uSFR was associated with lower odds of reporting halitosis (OR = 0.41, 95% CI 0.24–0.68, *p* < 0.001), reinforcing the protective association of preserved salivary function. Conversely, each unit increase in TCI more than doubled the odds (OR = 2.13, *p* = 0.002), and each 50-ppb rise in instrumental VSC raised them by 48% (*p* < 0.001), establishing a close link between chemical load and conscious perception. Polypharmacy emerged as an independent risk marker (OR = 2.87, 95% CI 1.18–6.97, *p* = 0.020) even after adjustment for uSFR and biofilm metrics, suggesting that medication burden may capture additional pathways or residual comorbidity. Mouth breathing tripled the odds of self-reported halitosis (OR = 3.42, *p* = 0.015), making it one of the most actionable risk markers in the table. Complete denture wearing carried roughly four-fold the odds compared with fixed prosthesis wearing (OR = 3.96, *p* = 0.026), whereas partial denture wearing did not reach significance after adjustment, indicating that the most distinct risk profile attaches to full edentulism with removable prostheses rather than to denture wearing in general. Each five-tooth increment in remaining natural dentition reduced odds by 17% (*p* = 0.040). Given the modest events-per-variable ratio, these estimates require confirmation in larger datasets.

Table 7 shows the central statistical interaction identified in the study: polypharmacy and prosthesis type were not only additively associated with VSC output, but their association differed by rehabilitation group. The Panel A breakdown reveals a group-dependent polypharmacy difference—a near-zero −5.5 ppb difference among complete denture wearers (*p* = 0.726) compared with +53.5 ppb in partial denture wearers (*p* = 0.001) and +57.5 ppb in fixed prosthesis/implant wearers (*p* < 0.001). A hypothesis-generating biological interpretation is one of possible ceiling saturation: complete denture wearers may already harbor extensive odorigenic biofilm reservoirs in the prosthesis–mucosa interface, so additional xerogenic medication burden may have limited measurable room to increase the substrate–enzyme cycle. In contrast, fixed prosthesis wearers had lower baseline VSC values, so reduced salivary flow associated with medication burden may be more visible. Panel B formalizes this asymmetric pattern statistically: the prosthesis main effect explains 52.2% of variance partial η^2^ (η^2^*_p_*), the polypharmacy main effect 23.5%, and the interaction term a smaller but significant 8.2% (F = 3.74, *p* = 0.029). These estimates should be interpreted as cross-sectional associations rather than causal effects.

Coefficients are unstandardized. uSFR scaled in mL·min^−1^; outcome is log_10_-VSC. Sobel z = 3.50 supports a statistically significant indirect association. Covariates included: age, prosthesis type, and mouth breathing. Because all variables were measured cross-sectionally, this analysis does not establish temporal mediation or causality. The primary covariate set was intentionally limited to age, prosthesis type, and mouth breathing to avoid overadjusting for oral biofilm variables.

Table 8 presents an exploratory statistical decomposition of the association between polypharmacy and halitosis-related VSC. The total association (path c) of polypharmacy with log_10_-VSC was 0.137 (*p* < 0.001), corresponding to roughly a 37% higher absolute VSC level among patients on ≥5 medications. When uSFR was added as a statistical mediator, the direct association (path c′) decreased to 0.074 (*p* = 0.006) but did not disappear, yielding a significant residual component and findings consistent with partial—rather than full—indirect association. The indirect association (a × b) was 0.063 with a bootstrap 95% confidence interval of 0.029 to 0.099, excluding zero, and the Sobel z of 3.50 (*p* < 0.001) provided concordant inference. Overall, 45.9% of the observed polypharmacy–VSC association was statistically explained by lower salivary flow (bootstrap 95% CI 21.6–67.8%); however, because of the cross-sectional design, this estimate should not be interpreted as proof that polypharmacy temporally reduced uSFR or that reduced uSFR causally transmitted the effect of polypharmacy. When TCI and denture biofilm/plaque were added to the mediation covariates, the indirect association through uSFR remained statistically significant but was attenuated (a × b = 0.047, 95% CI 0.018–0.083; *p* = 0.006), indicating that salivary flow did not merely proxy for tongue coating or prosthesis biofilm burden.

Overall, the sensitivity analyses supported the stability of the main findings. Excluding implant-supported restorations addressed the concern that the fixed/implant category might have included patients affected by a recent implant-related healing period, while excluding MMSE 24–26 participants addressed the reliability of self-reported halitosis. The expanded mediation model demonstrated that the salivary-flow indirect association persisted after accounting for tongue coating and denture biofilm/plaque, and the xerogenic medication analysis showed that the medication burden signal was not merely an artifact of using the standard ≥5-medication polypharmacy threshold. Importantly, the xerogenic count mediation proportion of 41.2% is now accompanied by its bootstrap 95% CI (19.4–62.7%), preventing the estimate from being presented as a single unsupported point value. In the MMSE exclusion model, protective and risk marker directions were preserved: higher uSFR remained associated with lower odds of self-perceived halitosis, whereas higher TCI, polypharmacy, mouth breathing, total VSC, and complete denture status remained associated with higher odds. 

Table 9 translates the predictive results into the practical language of bedside screening, providing operating characteristics that a clinician can interpret without further computation. Total VSC at the Youden optimal threshold of 210 ppb yielded sensitivity of 78.6% and specificity of 76.1%, with a positive predictive value of 75.0% in the observed prevalence setting (47.7%). The single best individual cut point was the organoleptic score ≥ 2.5, which delivered a sensitivity of 76.2% with the highest specificity among single predictors (82.6%) and a positive likelihood ratio of 4.38, sufficient to meaningfully shift post-test probability. uSFR ≤ 0.21 mL·min^−1^ and TCI ≥ 1.95 each performed in the moderate range, with sensitivities and specificities clustered around 70%, suggesting limited utility as standalone tests but potential value as components of a composite indicator. Indeed, the composite score (counting how many of four binary criteria—high VSC, low uSFR, heavy tongue coating, polypharmacy—were present) substantially outperformed any single component: at a threshold of ≥3 of 4, sensitivity reached 85.7% with specificity 78.3% (LR+ = 3.95, LR− = 0.18), making it a strong rule-out tool for ambulatory geriatric clinics. At the more stringent threshold of ≥4 of 4, specificity climbed to 93.5% and LR+ to 9.86, making it a strong rule-in tool when high specificity is required. To avoid ambiguity, the apparent AUC of 0.92 refers to the full multivariable logistic model in Table 6, whereas the apparent AUC of 0.89 refers to the simplified four-item chairside composite model described here. Neither AUC was optimism-corrected or externally validated. These values should therefore be read as apparent in-sample discrimination rather than validated clinical performance estimates. Table 10 reports the missing bootstrap 95% CI for the xerogenic count proportion explained and gives the coefficient direction for the MMSE exclusion halitosis model.

## 4. Discussion

### 4.1. Analysis of Findings

This study’s most clinically distinctive finding is the prosthesis × polypharmacy interaction: polypharmacy was associated with little difference among complete denture wearers (−5.5 ppb) but with 53–58 ppb higher VSC among partial denture and fixed prosthesis wearers, who together account for nearly two-thirds of the cohort. A plausible interpretation is a saturation pattern—complete denture wearers already host a heavy odorigenic biofilm in the prosthesis–mucosa interface [20,28], so additional xerogenic burden may have limited measurable room to increase the substrate ceiling. Tongue dorsum dysbiosis on the textured upper surface of complete dentures has been documented to reach bacterial densities comparable to those of severe periodontal disease [22], supporting this hypothesis. In contrast, fixed prosthesis wearers begin from lower VSC values (mean 143 ppb when not on polypharmacy), and the association with xerogenic medication burden is therefore more visible, a pattern compatible with observations in xerostomia cohorts [23]. Clinically, these findings suggest that deprescribing review and salivary-support strategies may be particularly relevant for dentate or fixed prosthesis elderly patients, whereas biofilm-targeted measures (mechanical denture cleaning, antimicrobial soaks, daily tongue scraping) remain essential for patients with full removable rehabilitations [19]. This interpretation remains hypothesis-generating because prosthesis type was also associated with age, medication burden, functional dependence, and remaining teeth.

The mediation analysis adds a hypothesis-generating indirect association layer rather than causal proof. Approximately 46 percent of the observed polypharmacy–log_10_-VSC association was statistically explained through lower salivary flow (95% CI 21.6–67.8%), while the remaining component persisted after uSFR adjustment. Reduced uSFR is biologically plausible in this framework because saliva is the principal vehicle for clearing VSC precursors from the oral cavity [18,26]; without adequate flow, methyl-mercaptan and hydrogen sulfide may accumulate on poorly cleansed mucosa to concentrations that breach the olfactory threshold [24,25]. The residual association may reflect medication-specific pathways that bypass salivary mechanisms, including reflux-related effects, dysgeusia, changes in mucosal integrity, or shared substrates of polypharmacy and frailty such as poor diet and reduced functional capacity [27]. This dual-path interpretation converges with our finding that polypharmacy retained an adjusted odds ratio of 2.87 for self-perceived halitosis even after uSFR was included in the logistic model. Nevertheless, because all variables were measured at one time point, the analysis should be read as partial statistical explanation, not as evidence that salivary flow temporally mediated a causal polypharmacy effect.

**Figure 1:**
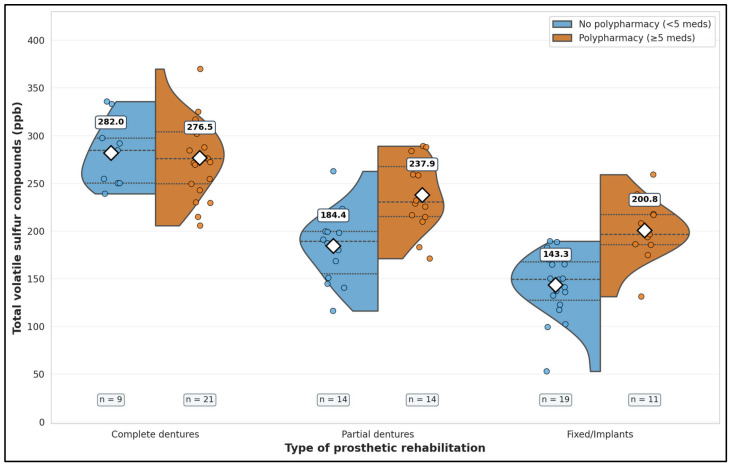
Distribution of total volatile sulfur compounds (VSCs) by prosthesis type and polypharmacy status (*n* = 88). Split-violin plots are overlaid with individual data points and cell mean diamonds. The polypharmacy-associated difference was −5.5 ppb in complete denture wearers, +53.5 ppb in partial denture wearers, and +57.5 ppb in fixed prosthesis/implant wearers. Two-way ANOVA identified a significant prosthesis × polypharmacy interaction (F = 3.74, *p* = 0.029, partial η^2^ = 0.082), interpreted as an effect modification pattern rather than causal proof.

**Figure 2:**
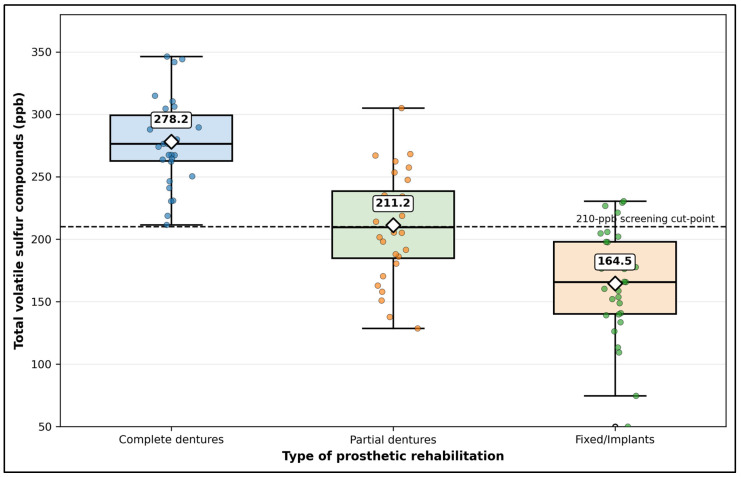
Boxplot of total volatile sulfur compounds (VSC) by prosthetic rehabilitation type. The boxplot illustrates the prosthesis-stratified VSC gradient observed in Table 3: complete dentures (278.2 ± 38.6 ppb), partial dentures (211.2 ± 46.3 ppb), and fixed/implant restorations (164.4 ± 43.9 ppb). Boxes represent interquartile ranges, whiskers 1.5 × IQR, points individual observations, and diamonds group means; the dashed horizontal line marks the 210 ppb screening cut point used in Table 9. One-way ANOVA showed a large between-group effect (F = 53.74, *p* < 0.001), with all Tukey pairwise comparisons significant.

**Figure 3:**
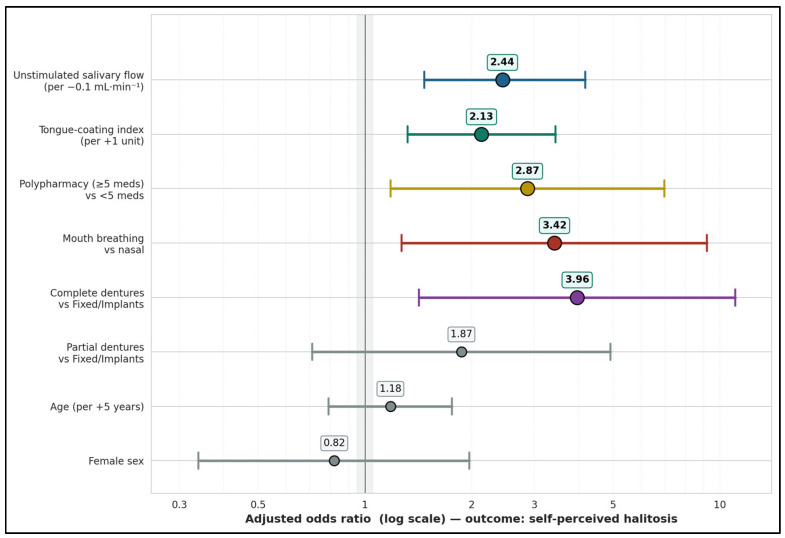
Forest plot of adjusted odds ratios from the multivariable logistic regression model for self-perceived halitosis (*n* = 88; Nagelkerke R^2^ = 0.58; Hosmer–Lemeshow *p* = 0.634). Each marker shows the point estimate with its 95% confidence interval. Significant risk markers included lower unstimulated salivary flow, higher tongue coating index, polypharmacy, mouth breathing, and complete denture wearing versus fixed/implant rehabilitation; partial denture status, age, and sex did not contribute additional independent information after adjustment. Because there were 42 outcome events and eight predictors, the model should be considered exploratory and internally unvalidated.

Our findings align with prior cross-sectional work in older adults [16,17,30] showing supragingival plaque and tongue coating as key correlates of halitosis, but extend that literature in three ways: (i) by stratifying explicitly on prosthesis type—the modal exposure variable in geriatric dental clinics; (ii) by formally testing the prosthesis × polypharmacy interaction, visible as a divergence of polypharmacy-associated VSC differences across rehabilitation types in Figure 1; and (iii) by using exploratory mediation to describe how much of the polypharmacy–VSC association was statistically explained by salivary hypofunction. At the same time, prosthesis type should be considered a rehabilitation context and clinical risk marker rather than an isolated causal determinant. Complete denture status in this cohort was accompanied by older age, heavier medication burden, greater functional dependence, fewer remaining teeth, and more mouth breathing, all of which may contribute independently to oral malodor. This interpretation is clinically useful because it encourages clinicians to treat prosthesis category as a signal for broader geriatric assessment rather than as a single mechanical explanation (Figure 2 and Figure 3). Nevertheless, these findings should be interpreted in light of potential residual confounding from unmeasured or incompletely controlled factors, including underlying comorbidities and other patient- and treatment-related characteristics [31]. Future longitudinal studies should stratify procedure type more granularly and include post-procedure time points earlier and later than four weeks.

### 4.2. Study Limitations

The cross-sectional design precludes causal inference; in particular, the observed mediation by uSFR rests on assumptions of correct path direction and no unmeasured confounders, neither of which can be definitively verified without longitudinal data. Baseline confounding across prosthesis groups is also important: complete denture wearers were older, had a heavier medication burden, more functional dependence, fewer remaining teeth, and higher mouth breathing prevalence than fixed prosthesis wearers. Although multivariable models adjusted for several of these factors, residual confounding is likely, and prosthesis type may function partly as a marker of broader oral and systemic frailty rather than an isolated causal exposure. The multivariable modeling should also be interpreted cautiously. Stepwise regression was avoided in the revised analysis narrative because of known concerns about unstable coefficients, inflated type I error, and reduced reproducibility, but the available sample still limited the number and stability of covariates that could be examined. Penalized approaches such as LASSO were not applied. Likewise, the logistic model included eight predictors for only 42 self-perceived halitosis events, giving an events-per-variable ratio of approximately 5.3; the apparent AUC values of 0.92 for the full model and 0.89 for the simplified composite may therefore be optimistic. No cross-validation, optimism correction, shrinkage, or external validation was performed. All predictive performance estimates (AUCs) were derived from the derivation sample without internal validation or optimism correction; therefore, they may overestimate real-world discrimination. For this reason, the chairside composite should be considered a hypothesis-generating screening aid until it is recalibrated and validated in an independent geriatric dental cohort. Convenience sampling at a single university outpatient clinic limits external validity: our patients were ambulatory, MMSE ≥ 24, and lived in the community, which excludes the far more vulnerable subset of frail or institutionalized elderly in whom denture hygiene and polypharmacy may behave differently. Halimeter measurements, while practical, quantify total sulfur load rather than compound-specific signatures and do not capture extra-oral halitosis. Finally, medication classes were recorded but not sufficiently powered for class-specific modeling, so the polypharmacy variable should be interpreted as an aggregate marker of medication burden and comorbidity.

Several additional sources of residual confounding should be acknowledged. First, the term “routine dental procedures” covered heterogeneous care pathways; we clarified that no patient was assessed during active implant osseointegration and added a conservative sensitivity analysis excluding implant-supported restorations, but the study was still not powered to compare each procedural subtype separately. Second, the four-week timing was selected to avoid immediate post-procedural effects and to reflect a practical follow-up visit, yet biofilm can re-establish within 2–7 days; results might differ at two-week or eight-week assessments. Third, only unstimulated whole saliva was collected. Stimulated flow was not available from this cohort or from pilot data, although it could better reflect chewing-related xerostomia complaints. Fourth, TCI was a strong predictor, but tongue cleaning behavior was not recorded, and denture adhesive use or effervescent/chemical cleaning solution type was not captured systematically. These factors may modify tongue coating, denture biofilm, and VSC production and should be incorporated into future prospective protocols.

## 5. Conclusions

The principal novel finding is that polypharmacy and prosthesis type showed a statistical interaction rather than a simple additive pattern: complete denture wearers showed essentially no incremental VSC difference by polypharmacy status, while partial denture and fixed prosthesis wearers showed substantially higher mean VSC values in the presence of polypharmacy. The exploratory mediation analysis indicated that roughly 46% of the polypharmacy–VSC association was statistically explained by lower salivary flow, but the cross-sectional design prevents causal or temporal interpretation. For clinicians, the combined assessment of prosthesis type, medication burden, salivary flow, tongue coating, and mouth breathing may help identify elderly patients who warrant targeted hydration counseling, tongue cleaning, denture hygiene reinforcement, and medication review. The full multivariable model had an apparent AUC of 0.92, whereas the simplified four-item chairside composite had an apparent AUC of 0.89; both estimates require optimism correction and external validation before use as definitive predictive tools. The updated sensitivity reporting further supports that the main predictor directions persisted after excluding lower MMSE participants and that the xerogenic count mediation estimate includes appropriate bootstrap uncertainty. The added sensitivity analyses suggest that these associations were not explained solely by implant-supported restorations, lower-but-eligible MMSE scores, or omission of TCI and biofilm/plaque from the primary mediation covariate set.

## Figures and Tables

**Table 1:** Baseline demographic, clinical, and oral health characteristics by prosthesis group (*n* = 88).

Variable	Complete Dentures (*n* = 30)	Partial Dentures (*n* = 28)	Fixed/Implants (*n* = 30)	*p*-Value
Age (years), mean ± SD	76.4 ± 5.3	71.6 ± 4.8	69.7 ± 4.4	<0.001
Female, *n* (%)	17 (56.7)	14 (50.0)	14 (46.7)	0.745
BMI (kg/m^2^), mean ± SD	26.7 ± 3.8	27.2 ± 3.5	26.4 ± 3.2	0.612
Diabetes mellitus, *n* (%)	11 (36.7)	7 (25.0)	6 (20.0)	0.376
Number of chronic medications	6.3 ± 2.4	4.6 ± 2.1	3.4 ± 1.9	<0.001
Current smoking, *n* (%)	7 (23.3)	6 (21.4)	5 (16.7)	0.812
Number of xerogenic medications	2.2 ± 1.1	1.5 ± 0.9	1.1 ± 0.8	0.001
Polypharmacy (≥5 meds), *n* (%)	21 (70.0)	14 (50.0)	11 (36.7)	0.034
Mouth breathing during sleep, *n* (%)	12 (40.0)	7 (25.0)	4 (13.3)	0.043
Functional dependence (Katz ≤ 5), *n* (%)	9 (30.0)	4 (14.3)	2 (6.7)	0.039
Number of remaining teeth, mean ± SD	0	12.7 ± 4.3	22.6 ± 3.9	<0.001
uSFR (mL·min^−1^), mean ± SD	0.18 ± 0.07	0.23 ± 0.08	0.28 ± 0.09	<0.001
Tongue coating index (0–3)	2.3 ± 0.6	1.9 ± 0.5	1.4 ± 0.4	<0.001
Denture biofilm/plaque index	2.4 ± 0.6	1.7 ± 0.4	1.2 ± 0.3	<0.001
Total VSC (ppb), mean ± SD	278.2 ± 38.6	211.2 ± 46.3	164.4 ± 43.9	<0.001
Organoleptic score (0–5)	3.4 ± 0.7	2.7 ± 0.6	1.9 ± 0.7	<0.001
GOHAI score (12–60)	39.7 ± 6.4	47.3 ± 5.8	53.6 ± 4.7	<0.001
Self-perceived halitosis, *n* (%)	22 (73.3)	13 (46.4)	7 (23.3)	<0.001

**Table 2:** Spearman correlation coefficients (ρ) among principal continuous variables (*n* = 88).

Variable	uSFR	TCI	Biofilm/Plaque	Medications	Total VSC
uSFR	—	−0.49 ***	−0.36 **	−0.42 ***	−0.61 ***
TCI	−0.49 ***	—	0.41 ***	0.34 **	0.56 ***
Biofilm/plaque	−0.36 **	0.41 ***	—	0.27 *	0.47 ***
# Medications	−0.42 ***	0.34 **	0.27 *	—	0.49 ***
Remaining teeth	0.31 **	−0.38 **	−0.43 ***	−0.29 *	−0.39 **
Age (years)	−0.27 *	0.24 *	0.18 (NS)	0.36 **	0.31 **
GOHAI score	0.34 **	−0.41 ***	−0.39 **	−0.32 **	−0.51 ***
Organoleptic	−0.52 ***	0.43 ***	0.39 **	0.41 ***	0.71 ***

* *p* < 0.05, ** *p* < 0.01, *** *p* < 0.001 (Bonferroni-corrected for multiple comparisons). NS = not significant.

**Table 3:** Mean values of objective halitosis markers by prosthesis group, with one-way ANOVA and Tukey HSD post hoc comparisons.

Outcome	Complete Dentures (A)	Partial Dentures (B)	Fixed/Implants (C)	F (df 2, 85)	*p*-Value
Total VSC (ppb)	278.2 ± 38.6	211.2 ± 46.3	164.4 ± 43.9	53.74	<0.001
log_10_-VSC	2.44 ± 0.06	2.32 ± 0.10	2.21 ± 0.12	47.62	<0.001
Organoleptic (0–5)	3.4 ± 0.7	2.7 ± 0.6	1.9 ± 0.7	37.18	<0.001
TCI (0–3)	2.3 ± 0.6	1.9 ± 0.5	1.4 ± 0.4	23.61	<0.001
uSFR (mL·min^−1^)	0.18 ± 0.07	0.23 ± 0.08	0.28 ± 0.09	12.34	<0.001

Tukey post hoc tests: all pairwise comparisons were statistically significant for total VSC, log_10_-VSC, organoleptic score, and TCI (A vs. B: *p* < 0.001; A vs. C: *p* < 0.001; B vs. C: *p* ≤ 0.001). For uSFR, A vs. C *p* < 0.001, A vs. B *p* = 0.041, B vs. C *p* = 0.038. Cohen’s d for VSC: A vs. C = 2.74; A vs. B = 1.56; B vs. C = 1.04 (all interpreted as large effects).

**Table 4:** Theory-informed multivariable linear regression predicting log_10_-transformed total VSC (*n* = 88).

Predictor	β (Std)	SE (β)	95% CI	t-Value	*p*-Value
uSFR (per 0.1 mL·min^−1^)	−0.46	0.07	−0.60 to −0.32	−6.57	<0.001
Tongue coating index	0.28	0.06	0.16 to 0.40	4.67	<0.001
Polypharmacy (≥5 meds)	0.21	0.07	0.07 to 0.35	3.00	0.004
Mouth breathing (yes)	0.16	0.07	0.02 to 0.30	2.29	0.025
Number of remaining teeth	−0.13	0.06	−0.25 to −0.01	−2.17	0.033
Denture biofilm/plaque	0.14	0.07	0.00 to 0.28	2.00	0.049
Age (per 5 years)	0.08	0.06	−0.04 to 0.20	1.33	0.187
Sex (male vs. female)	0.04	0.07	−0.10 to 0.18	0.57	0.571

Adjusted R^2^ = 0.62; F(8, 79) = 18.94, *p* < 0.001; SE = 0.13 log units. Variance inflation factors all <1.8 (multicollinearity excluded). Age and sex were retained because of clinical relevance but were not statistically significant in the adjusted model.

**Table 5:** Subgroup analysis: halitosis indicators stratified by sleep mouth breathing and (in denture wearers) by denture cleaning compliance.

Outcome	Mouth Breathers (*n* = 23)	Nasal Breathers (*n* = 65)	*p*-Value	Daily Denture Cleaning (*n* = 27)	<Daily Denture Cleaning (*n* = 31)	*p*-Value
uSFR (mL·min^−1^)	0.17 ± 0.06	0.25 ± 0.09	<0.001	0.22 ± 0.08	0.19 ± 0.07	0.083
TCI (0–3)	2.4 ± 0.5	1.7 ± 0.6	<0.001	1.8 ± 0.5	2.3 ± 0.6	0.002
Denture biofilm/plaque	2.3 ± 0.7	1.6 ± 0.6	<0.001	1.6 ± 0.4	2.7 ± 0.5	<0.001
Total VSC (ppb)	262.7 ± 51.4	201.4 ± 56.8	<0.001	218.4 ± 48.7	274.6 ± 53.4	<0.001
Organoleptic (0–5)	3.3 ± 0.8	2.4 ± 0.8	<0.001	2.6 ± 0.7	3.3 ± 0.6	<0.001
Self-perceived halitosis, *n* (%)	16 (69.6)	26 (40.0)	0.014	12 (44.4)	23 (74.2)	0.022

**Table 6:** Multivariable logistic regression for self-perceived morning halitosis (dependent = “Yes”, *n* = 88).

Predictor	Adjusted OR	95% CI	Wald χ^2^	*p*-Value
uSFR (per 0.1 mL·min^−1^ increase)	0.41	0.24 to 0.68	11.62	<0.001
TCI (per 1-unit increase)	2.13	1.31 to 3.46	9.29	0.002
Polypharmacy (yes vs. no)	2.87	1.18 to 6.97	5.43	0.020
Mouth breathing (yes vs. no)	3.42	1.27 to 9.21	5.93	0.015
Total VSC (per 50-ppb increase)	1.48	1.21 to 1.81	13.84	<0.001
Prosthesis: complete (vs. fixed)	3.96	1.18 to 13.27	4.95	0.026
Prosthesis: partial (vs. fixed)	1.74	0.51 to 5.93	0.78	0.378
Number of remaining teeth (per 5)	0.83	0.69 to 0.99	4.21	0.040

**Table 7:** Two-way ANOVA: prosthesis type × polypharmacy on log_10_-VSC, with cell means and simple effect analyses (*n* = 88).

Panel A. Cell means (back-transformed VSC, ppb) and the marginal differences attributable to polypharmacy					
**Prosthesis Group**	**No Polypharmacy (<5 meds)**		**Polypharmacy (≥5 meds)**	**Δ VSC (Poly—No Poly)**	**Simple Effect *p***
Complete dentures	282.0 ± 36.2 (*n* = 9)		276.5 ± 40.3 (*n* = 21)	−5.5 ppb	0.726
Partial dentures	184.4 ± 38.3 (*n* = 14)		237.9 ± 37.9 (*n* = 14)	+53.5 ppb	0.001
Fixed/Implants	143.3 ± 34.4 (*n* = 19)		200.8 ± 33.8 (*n* = 11)	+57.5 ppb	<0.001
Panel B. Two-way ANOVA decomposition of variance in log_10_-VSC					
**Source of Variation**	**df**	**Mean Square**	**F**	***p*-Value**	**η^2^*_p_***
Prosthesis type (main effect)	2	0.348	44.62	<0.001	0.522
Polypharmacy (main effect)	1	0.196	25.13	<0.001	0.235
Prosthesis × polypharmacy interaction	2	0.029	3.74	0.029	0.082
Error (within cells)	82	0.0078	—	—	—
Total (corrected)	87	—	—	—	—

Adjusted R^2^ for the full two-way model = 0.65. Simple effect tests in Panel A used Bonferroni-corrected α = 0.0167.

**Table 8:** Exploratory mediation analysis: salivary flow as a statistical mediator of the polypharmacy–log_10_-VSC association (5000 bootstrap resamples, *n* = 88).

Effect Path	Estimate (β)	Bootstrap SE	95% Bias-Corrected CI	z-Value	*p*-Value
a path: polypharmacy → uSFR	−0.062	0.014	−0.091 to −0.034	−4.43	<0.001
b path: uSFR → log_10_-VSC (controlling for poly)	−1.23	0.21	−1.66 to −0.81	−5.86	<0.001
c path: total effect polypharmacy → log_10_-VSC	0.137	0.029	0.080 to 0.194	4.72	<0.001
c′ path: direct effect (with uSFR controlled)	0.074	0.027	0.021 to 0.127	2.74	0.006
a × b: indirect (mediated) effect	0.063	0.018	0.029 to 0.099	3.50	<0.001
Proportion of total effect mediated	45.9%	—	21.6% to 67.8%	—	—

**Table 9:** Diagnostic performance of clinical cut points for screening self-perceived halitosis (*n* = 88; 42 cases, 46 controls).

Predictor (Youden Optimal Cut Point)	Sensitivity (%)	Specificity (%)	PPV (%)	NPV (%)	LR+	LR−
Total VSC ≥ 210 ppb	78.6	76.1	75.0	79.5	3.29	0.28
uSFR ≤ 0.21 mL·min^−1^	73.8	67.4	67.4	73.8	2.27	0.39
TCI ≥ 1.95	71.4	69.6	68.2	72.7	2.35	0.41
Organoleptic score ≥ 2.5	76.2	82.6	79.5	79.6	4.38	0.29
Composite score ≥ 3 (any 4) *	85.7	78.3	78.3	85.7	3.95	0.18
Composite score ≥ 4 (any 4) *	64.3	93.5	90.0	74.1	9.86	0.38

PPV/NPV calculated for the observed prevalence of 47.7%. * Composite score = number of the following present: VSC ≥ 210 ppb, uSFR ≤ 0.21 mL·min^−1^, TCI ≥ 1.95, polypharmacy. Cut points were selected by Youden index from ROC curves; bootstrap 95% CIs available on request. LR = likelihood ratio.

**Table 10:** Sensitivity analyses addressing procedural heterogeneity, cognitive status, covariate selection, and xerogenic medication burden.

Sensitivity Analysis	Analysis Set/Model	Results	Interpretation
Excluding implant-supported restorations	*n* = 76 after excluding 12 implant-supported crown/restoration cases; fixed group limited to tooth-supported fixed prostheses	Prosthesis × polypharmacy interaction retained: F = 3.38, *p* = 0.039; partial η^2^ = 0.081	The interaction was not driven by implant-supported restorations or a recent osseointegration effect.
Excluding mild cognitive impairment range	*n* = 78 after excluding 10 patients with MMSE 24–26	Self-perceived halitosis model remained directionally unchanged; prosthesis × polypharmacy interaction F = 3.31, *p* = 0.042; key ORs remained directionally similar after MMSE exclusion (uSFR 0.43 vs. 0.41; TCI 2.06 vs. 2.13; polypharmacy 2.72 vs. 2.87; complete dentures vs. fixed 3.81 vs. 3.96)	The main inference was robust to exclusion of participants with lower-but-eligible cognitive scores.
Expanded mediation covariates	Primary mediation covariates plus TCI and denture biofilm/plaque	Indirect association through uSFR remained significant: a × b = 0.047, 95% CI 0.018–0.083; *p* = 0.006; proportion explained = 37.6%; bootstrap 95% CI for proportion explained = 15.1–58.8%	Salivary flow was not simply a surrogate for tongue coating or prosthesis biofilm.
Xerogenic medication count instead of binary polypharmacy	Continuous count of medications with recognized xerogenic potential substituted for ≥5-medication status	Indirect association through uSFR remained significant: a × b = 0.021 per xerogenic medication, 95% CI 0.007–0.039; *p* = 0.009; proportion explained = 41.2%; bootstrap 95% CI for proportion explained = 19.4–62.7%	Medication-specific xerogenic burden gave comparable inference to the standard binary polypharmacy definition.

MMSE = Mini-Mental State Examination; TCI = tongue coating index; uSFR = unstimulated salivary flow rate; VSC = volatile sulfur compounds. Sensitivity analyses were post hoc and should be interpreted as robustness checks rather than confirmatory subgroup tests. The bootstrap 95% CIs for the proportions explained were generated from the empirical bootstrap distribution of the indirect/total-effect ratio and are reported to make Table 10 consistent with the primary mediation model.

## Data Availability

The data presented in this study are available on reasonable request from the corresponding authors. The dataset is not publicly available due to privacy and ethics restrictions related to identifiable geriatric clinical information.
